# Exploring the Reactivity of Na[W_2_(μ-Cl)_3_Cl_4_(THF)_2_]∙(THF)_3_ towards the Polymerization of Selected Cycloolefins

**DOI:** 10.3390/molecules201219810

**Published:** 2015-12-08

**Authors:** Nikolaos Saragas, Georgios Floros, Grigorios Raptopoulos, Marinos Pitsikalis, Patrina Paraskevopoulou, Konstantinos Mertis

**Affiliations:** 1 Department of Inorganic Chemistry, Faculty of Chemistry, University of Athens, Panepistimiopolis Zografou, Athens 15771, Greece; nsaragas@sch.gr (N.S.); geofloros@chem.uoa.gr (G.F.); grigorisrap@chem.uoa.gr (G.R.); 2 Department of Industrial Chemistry, Faculty of Chemistry, University of Athens, Panepistimiopolis Zografou, Athens 15771, Greece; pitsikalis@chem.uoa.gr

**Keywords:** metathesis, ROMP, metal-metal bonds, tungsten

## Abstract

The bimetallic compound Na[W_2_(μ-Cl)_3_Cl_4_(THF)_2_]·(THF)_3_ (**1**, {W^ 3 ^W}^6+^, *a′*^2^*e′*^4^) is a highly efficient room-temperature initiator for ring opening metathesis polymerization (ROMP) of norbornene (**NBE**) and some of its derivatives. In most cases, addition of phenylacetylene (**PA**) as co-initiator improves the catalytic activity and retains the high *cis*-stereoselectivity. On the other hand, **1** can polymerize cyclopentadiene (**CPD**), not via a metathetic, but rather, via a cationic mechanism. Here, we present a comparison of the reactivity of the two catalytic systems (**1** and **1**/**PA**) between themselves and with other systems reported in the literature, the characterization of the polymers formed and mechanistic aspects of the corresponding reactions.

## 1. Introduction

**Scheme 1:**
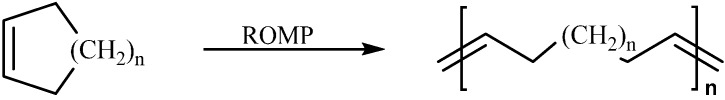
Ring opening metathesis polymerization (ROMP).

Olefin metathesis reactions are metal-mediated carbon-carbon (C–C) double-bond exchange processes with numerous important applications. Olefin metathesis polymerization is an application of metathesis reactions to polymer synthesis, which includes, among others, the ring opening metathesis polymerization (ROMP) process (Scheme 1). The latter provides a wide range of unsaturated polymers of unique architectures and useful functions (e.g., outstanding elastomeric or thermoplastic engineering materials), whose physicochemical properties depend strongly on their structure. The reaction can be catalyzed by a broad range of uni-, bi- or multi-component systems based on transition metal complexes (Ti, Nb, Ta, Cr, Mo, W, Re, Co, Ru, Os) [1,2,3,4,5,6], with those of Mo, W and Ru playing a major role. They can be classified into two major categories [1,2,3,4,5,6,7,8,9]: (i) ill-defined systems, whereas the active metallocarbenes are generated *in situ*; examples include: (a) the classical high-valent halides of Mo and W (e.g., WCl_6_ and derivatives thereof), which become very effective catalysts when activated by organometallic co-catalysts (e.g., SnMe_4_, AlEt_3_), and (b) the RuCl_3_/alcohol [10] catalytic system, which is used industrially; and (ii) well-defined metallocarbenes, such as the Grubbs [11] and Schrock [12] catalysts and their numerous variations.

Although ROMP was discovered in the mid-1950s and has been the subject of intensive research ever since, many difficulties are still encountered. For example, the Mo and W halides widely employed in industry present a number of drawbacks: (a) the halides themselves are hydrolytically unstable; (b) in some cases, high activity is achieved only with the use of organometallic initiators; (c) the polymerization is not “living”, preventing precise control over the reaction(s); (d) they are not stereoselective; and (e) the exact nature of the active species remains uncertain.

In contrast, precision catalysts guarantee a high degree of reaction control over a diverse range of cycloolefins. Considerable progress has been made with Grubbs catalysts in terms of stability and design, but they are very expensive and difficult to remove from the product. On the other hand, Schrock catalysts are highly reactive (especially the Mo-alkylidenes), but also highly sensitive to oxygen and moisture, and their synthesis is elaborate. Those issues present themselves as limitations for the utilization of those systems at a large scale.

Additionally, another long-standing problem is controlling the stereochemistry of the reaction (*cis*/*trans*). This issue has been overcome up to now by serendipity, empirically (additives affecting the stereochemistry of the reaction) or by elaborate catalyst design. Thus, improving known catalytic systems or finding new ones, which are robust, cost effective, highly active and stereoselective, is still a challenge.

The majority of existing catalytic systems consists of mononuclear complexes. Bimetallic complexes with metal-metal bonds have been scarcely employed [13], even though they provide more precise control over stereoselectivity via the involvement of both metal centers in the reaction. Among those, the ditungsten complex Na[W_2_(μ-Cl)_3_Cl_4_(THF)_2_]·(THF)_3_ (**1**, {W^ 3 ^W}^6+^, *a′*^2^*e′*^4^) is a highly efficient unicomponent room temperature homogeneous and/or heterogeneous initiator for the ROMP of norbornene (**NBE**) and some of its derivatives [14]. **1** distinctly differs from its mononuclear counterparts, offering significant advantages over them, such as: (a) high reactivity similar to that of the bi- or multi-component analogues (e.g., WCl_6_/AlR_3_); (b) high *cis*-stereoselectivity; and (c) tolerance to olefinic side groups, providing polymeric materials suitable for post-polymer functionalization.

In view of those properties of **1**, which may address some of the limitations previously indicated, and our continuing interest for developing robust and efficient catalytic systems, we have utilized phenylacetylene (**PA**) as co-initiator of the ROMP process with **1**, and we have examined the reactivity of **1**/**PA** towards a number of cycloolefins. In most cases, use of **PA** improves the catalytic activity of **1**, while the high *cis*-stereoselectivity is preserved. This paper presents a comparison of the reactivity of the two catalytic systems (**1** and **1**/**PA**) with themselves and with other relevant systems from the literature, as well as the characterization of the polymers formed and mechanistic aspects of the reactions.

## 2. Results and Discussion

### 2.1. Catalyst and Polymerization Reactions

Compound **1** (Supplementary Material Scheme S1) features a face-sharing dioctahedral fsbo geometry and contains a triple metal-metal bond. Structural characterization has revealed the presence of two THF ligands (one to each tungsten atom) in a *cis* arrangement along the dimetal axis [15], which is the key for the reactivity of this compound towards metathesis polymerization reactions [14,16]. Such species in solution, in the presence of donor ligands or coordinating solvents, may exist in an equilibrium between the highly symmetric confacial (*D*_3h_, fsbo) **1** and the edge-sharing biooctahedral (D_2h_, esbo) **1′** (Supplementary Material Scheme S1) [17]. In solution, **1** is air-sensitive (oxygen, moisture), but in the solid state, it is stable in air at room temperature for 1–2 h. It is soluble in THF, CH_3_CN and dimethoxyethane (dme), less soluble in CH_2_Cl_2_ and CHCl_3_ and insoluble in toluene and Et_2_O. It was repeatedly recrystallized and checked carefully for purity (UV-VIS) before use. Polymerization reactions were carried out at room temperature, for a given time, *t*. Results are summarized in Table 1. For comparison purposes, Table 1 also includes published data of **1**-catalyzed ROMP of cycloolefins [14]. All possible reaction pathways and products are shown in Supplementary Material Scheme S2.

**Table 1:** Polymerization of cycloolefins with the catalytic systems **1** and **1**/phenylacetylene (**PA**). dme, dimethoxyethane; **NBE**, norbornene; **VNBE**, 5-vinyl-2-norbornene; **NBE-COOMe**, methyl 5-norbornene-2-carboxylate; **NBE-CN**, 5-norbornene-2-carbonitrile; **NBE-EN**, 5-ethylidene-2-norbornene; **NBE-SiM**, 5-trimethoxysilyl-2-norbornene; **NBE-SiE**, 5-triethoxysilyl-2-norbornene; **NBD**, norbornadiene; **DCPD**, dicyclopentadiene.

Entry	Monomer	Solvent	1/PA/Monomer Molar Ratio	*t* (h)	Yield (%)	*M*_w_ × 10^−3 g^	*M*_w_/*M*_n_^g^
1	**NBE** [14]	THF	1/0/500 ^a^	19	12	86.2	1.2
2		CH_2_Cl_2_		1	96	529	1.2
3		dme		48	- ^f^	-	-
4		CH_3_CN		48	- ^f^	-	-
5		toluene		24	37	296	2.9
6		Et_2_O		20	94	422	1.4
7	**NBE/PA**	THF	1/20/500 ^b^	20	>99	184	1.5
8		CH_2_Cl_2_		0.1	>99	413	1.3
9		dme		20	18	3.2	1.5
10		CH_3_CN		48	- ^f^	-	-
11		toluene		20	34	11	1.5
12		Et_2_O		20	72	392	1.4
13	**NBE/PA**	THF	1/20/1000 ^c^	1	95	22.3	2.5
14		CH_2_Cl_2_		0.1	97	300	1.2
15		toluene		0.3	95	62	4.5
16	**VNBE** [14]	CH_2_Cl_2_	1/0/500 ^a^	8	>99	974	2.6
17	**VNBE/PA**	CH_2_Cl_2_	1/20/500 ^b^	1	>99	97	2.7
18	**NBE-COOMe** [14]	CH_2_Cl_2_	1/0/500 ^a^	12	>99	685	1.15
19	**NBE-COOMe/PA**	CH_2_Cl_2_	1/20/500 ^b^	6	90	- ^h^	-
20		-	1/20/1000 ^c^	1	40	810	1.2
21	**NBE-CN** [14]	CH_2_Cl_2_	1/0/500 ^a^	48	- ^g^	-	-
22	**NBE-CN/PA**	CH_2_Cl_2_	1/20/500 ^b^	6	17	- ^i^	-
23		-	1/20/1000 ^c^	20	30	- ^i^	-
24	**NBE-EN**	CH_2_Cl_2_	1/0/500 ^a^	0.3	97	452	1.3
25	**NBE-EN/PA**	CH_2_Cl_2_	1/20/500 ^b^	4	>99	583	1.2
26		-	1/20/1000 ^c^	20	>99	974	2.6
27	**NBE-SiM**	CH_2_Cl_2_	1/0/500 ^a^	20	60	- ^i^	-
28	**NBE-SiE**	CH_2_Cl_2_	1/0/500 ^a^	20	60	- ^i^	-
29	**NBE-SiE/PA**	CH_2_Cl_2_	1/20/500 ^b^	8	75	- ^i^	-
30	**NBD** [14]	THF	1/0/500 ^a^	4	>99	- ^j^	-
31	**NBD/PA**	THF	1/20/500 ^b^	0.5	38	- ^j^	-
32			1/20/1000 ^c^	21	61	- ^j^	
33	**DCPD**	CH_2_Cl_2_	1/0/500 ^a^	17	10	- ^j^	-
34		toluene		17	20	- ^j^	-
35		-		17	20	- ^j^	-
36	**DCPD/PA**	CH_2_Cl_2_	1/20/500 ^b^	17	>99	- ^j^	-
37		toluene		17	>99	- ^j^	-
38		-	1/20/1000 ^c^	20	traces	-	-
39	**CPD**	CH_2_Cl_2_	1/0/250 ^d^	20	90	29.7	2.1
40		THF		20	5	-	-
41		toluene		20	30	3.3	1.5
42		-		20	30	6.3	2.7
43	**CPD/PA**	CH_2_Cl_2_	1/10/250 ^e^	8	50	11.1	1.3
44		toluene		8	25	1.2	1.6

^a^ Conditions: **1** (9.0 mg, 0.009 mmol), monomer (4.5 mmol)/5.0 mL solvent; ^b^ conditions: **1** (9.0 mg, 0.009 mmol), **PA** (20 μL, 18.4 mg, 0.18 mmol), monomer (4.5 mmol)/5.0 mL solvent; ^c^ conditions: **1** (9.0 mg, 0.009 mmol), **PA** (20 μL, 18.4 mg, 0.18 mmol), monomer (9.0 mmol)/5.0 mL solvent or bulk; ^d^ conditions: **1** (9.0 mg, 0.009 mmol), cyclopentadiene (**CPD**) (225 μL, 178 mg, 2.7 mmol)/2.0 mL solvent; ^e^ conditions: **1** (9.0 mg, 0.009 mmol), **PA** (10 μL, 9.2 mg, 0.09 mmol), **CPD** (225 μL, 178 mg, 2.7 mmol)/2.0 mL solvent; ^f^ no polymerization; ^g^ by size exclusion chromatography (SEC) in THF at 40 °C *vs.* polystyrene standards; ^h^ molecular weight higher than 1,000,000; ^i^ molecular weight could not be determined because of the negative refraction index of the solution; ^j^ polymer insoluble in THF or DMF.

### 2.2. Ring Opening Metathesis Polymerization Reactions

The polymerization of norbornene (**NBE**) induced by **1** was previously studied, and it was found to proceed either homogeneously or heterogeneously in different solvent media (Table 1, Entries 1–6) [14]. In coordinating solvents, the system was either inactive (dimethoxyethane (dme), CH_3_CN) or afforded small yields of **PNBE** (THF, 12%, 19 h). In CH_2_Cl_2,_ gelation was fast (1 h), and the polymer was obtained in high yield (96%) and had very good molecular characteristics (*M*_w_ ≈ 529,000, *M*_w_/*M*_n_ = 1.2). Suspension of **1** in toluene gave moderate yields of **PNBE** (37%, 24 h, *M*_w_ ≈ 296,000, *M*_w_/*M*_n_ = 2.9), while suspensions in Et_2_O (94%, 20 h) gave high yields of high-molecular weight **PNBE** (*M*_w_ ≈ 422,000, *M*_w_/*M*_n_ = 1.4). The *cis*-stereoselectivity was high (86%) in all cases, and it was not affected by the reaction conditions.

Reactions with the catalytic system **1**/**PA** (Table 1, Entries 7–12) were run at the same **1**/**NBE** molar ratio and under the same reaction conditions. **PA** (**1**/**PA**/**NBE** 1/20/500) was added to the system prior to the addition of **NBE**. The best results were obtained in THF and CH_2_Cl_2_ (Entries 7 and 8). In both cases, the reactions were quantitative, providing polymers with high molecular weights and fairly narrow molecular weight distributions. By comparison to **1**, the catalytic system **1**/**PA** was significantly more active in those solvents. The rate of the reaction was significantly enhanced (in CH_2_Cl_2_, the reaction was completed within minutes), and yields were quantitative. The molecular weight of **PNBE** obtained in THF was doubled; this, in addition to the increase of the reaction yield, indicated good control over the polymerization reaction. In dme (Entry 9), low molecular weight polymer was obtained in low yield, while in CH_3_CN, the catalytic system was unreactive, even after long reaction times (Entry 10). In toluene (Entry 11), the rate of the reaction and the yield were not altered by the addition of **PA**, but the molecular weight of **PNBE** formed was significantly lower (by a factor of 27) and the molecular weight distribution much narrower. This can be explained by considering the solubility of the active intermediate in toluene: **1**/**PA** in toluene turned homogeneous very quickly, and therefore, the concentration of the active sites for polymerization was higher (compared to **1**), leading to a better-controlled polymerization reaction. On the other hand, in Et_2_O (Entry 12), in which the catalytic system remained heterogeneous during the course of the reaction, the molecular characteristics of **PNBE** obtained by the two systems were similar, while the yield was somewhat lower. The addition of **PA** to the catalytic system did not affect the *cis*-stereoselectivity of the reactions, as high *cis* (86%–88%) polymers were obtained in all cases.

When higher ratios of **NBE**/**1** were employed (1000/1; Entries 13–15), the reaction was accelerated, but polymers with lower molecular weights and broad molecular weight distributions were obtained, indicating that not only the main reaction, but also termination reactions were accelerated; and secondary metathesis reactions caused “chopping” of the polymeric chains, as was previously observed for catalytic System **1** [14,16]. This effect was more obvious in THF and toluene, for which the reaction times were higher.

All **PNBE** samples obtained were soluble in common organic solvents (CHCl_3_, CH_2_Cl_2_, THF). The configuration of the polymer was determined by ^1^H- and ^13^C-NMR spectra (Supplementary Material Figure S1) [18]. The relative proportions of double-bond pair sequences, represented as *trans-cis* (*tc*), *trans-trans* (*tt*), *cis-cis* (*cc*) and *cis-trans* (*ct*) units, were determined from the four methine carbon (C^1,4^) signals of the ^13^C-NMR spectrum of **PNBE** at δ_C_ 43.67 (*tc*), 43.44 (*tt*), 38.88 (*cc*) and 38.67 ppm (*ct*). The fraction of *cis* double bonds (σ_c_ = 0.85) estimated from this ^13^C-NMR spectrum was in good agreement with that obtained from the ^1^H-NMR spectrum (σ_c_ = 0.86) by integration of the signals at δ_H_ 2.73 (HC^1,4^ *cis-***PNBE**) and 2.37 ppm (HC^1,4^ *trans-***PNBE**). The reactivity ratios *r_c_* = *cc/ct* = 7.4, *r_t_* = *tt/tc* = 1.3 and *r_c_r_t_* = 9.6 were calculated from the heights of the relevant signals in the ^13^C-NMR spectra.

Other monomers that were activated by **1** were also studied with the catalytic system **1**/**PA**. These monomers include 5-vinyl-2-norbornene (**VNBE**), methyl 5-norbornene-2-carboxylate (**NBE-COOMe**), 5-norbornene-2-carbonitrile (**NBE-CN**), 5-ethylidene-2-norbornene (**NBE-EN**), 5-trimethoxysilyl-2-norbornene (**NBE-SiM**), 5-triethoxysilyl-2-norbornene (**NBE-SiE**), norbornadiene (**NBD**) and dicyclopentadiene (**DCPD**). Soluble polymers were characterized using NMR spectroscopy (Supplementary Material Figures S2–S3 and S5–S8).

**VNBE** and **NBE-COOMe** were polymerized quantitatively by **1** [14], yielding very high molecular weight polymers and, in the second case, a very narrow molecular weight distribution (Table 1, Entries 16 and 18). The addition of **PA** reduced the reaction time significantly, while reactions remained quantitative (**VNBE**; Entry 17) or almost quantitative (**NBE-COOMe**; Entry 19). **NBE-COOMe** was also activated in bulk (Entry 20); the reaction was fast, the molecular weight was high, and the molecular weight distribution was narrow, but the yield was moderate, due to gelation of the reaction mixture. The most notable change in reactivity was, again, the molecular weight of the polymers formed. For **PVNBE**, it was significantly lower (by a factor of 10), while for **PNBE-COOMe**, it increased so much that it could not be determined by size exclusion chromatography (SEC) accurately. The ^1^H-NMR spectrum of **PVNBE** (Supplementary Material Figure S2) indicated that the ring-strained C=C bond was cleaved, while the vinylic one was left intact. The same reactivity had been observed with **1** [14]. The overlapping signals of the olefinic protons of the polymeric chain with the vinylic ones prevented stereoregular assignment.

Both monomers are of interest, as their polymers can be easily functionalized by the addition of new side groups via reactions with the pendant vinyl bonds and acetate units. The pendant vinyl group of **VNBE** is usually involved in metathesis reactions, leading to cross-linked products, thus used for the synthesis of self-healing polymers [19]. There are only three more catalytic systems that polymerize **VNBE** in a manner similar to **1** and **1**/**PA**: (a) [(CO)_4_W(μ-Cl)_3_W(GeCl_3_)(CO)_3_] [20], yielding polymers of low molecular weight (<3000) and bimodal peaks of a broad molecular weight distribution (>2); (b) imidotungsten(VI) complexes bearing chelating phenol ligands, activated by EtMgBr, at room temperature, which provided polymers in moderate yields (up to 50%), but molecular characteristics of the samples have not been reported [21]; and (c) [V(CHSiMe_3_)(NAd)(OC_6_F_5_)(PMe_3_)_2_], giving quantitative yields at room temperature [22]. High and very high molecular weight polymers of **NBE-COOMe** have also been obtained by Ru-based catalytic systems, with yields varying from moderate to quantitative, but the molecular weight distributions were either not reported or were broad [23,24,25].

**NBD** was also polymerized quantitatively by **1** [14] in CH_2_Cl_2_, THF and toluene, as well as in bulk, yielding insoluble polymers. The reactions were completed within 5 min in all cases, except for THF, in which the rate of polymerization was much slower (Table 1, Entry 30). The addition of **PA** yielded moderate yields of insoluble **PNBD** within minutes (Entry 31). The reaction could not be kept longer, because of the gelation of the reaction mixture. Thermogravimetric analysis of the polymer obtained showed a decomposition peak at high temperature (455 °C; Supplementary Material Figure S4), indicating a high degree of crosslinking.

Substrates bearing strongly coordinating side groups (–COOH, –OH, –CN) were not polymerized by **1** in all solvents studied (CHCl_3_, CH_2_Cl_2_, THF, toluene) or in bulk [14]. With the catalytic system **1**/**PA**, only **NBE-CN** could be activated in CH_2_Cl_2_ or in bulk, providing **PNBE-CN** in low yields (Entries 22 and 23). That monomer can rarely be activated by ROMP catalytic systems [24,26], and when it does, the yields of **PNBE-CN** obtained are low, with only one exception (85% yield), when activated by a Ru-based catalytic system in ionic liquids [24]. The most probable reason for such low reactivity is that the –CN group interacts strongly with the metal active sites and, therefore, inhibits sustainable polymerization.

Another very interesting monomer is **NBE-EN**, which is used for the synthesis of self-healing homo- [27] or co-polymers (with **DCPD**) [28] using Ru-based catalysts. In contrast, Mo- [29] and V-based [22] catalysts provide soluble polymers. In the first case, **PNBE-EN** with *M*_n_ = 46,900 and a very narrow molecular weight distribution (<1.1) was obtained, while in the second case, the molecular weight was not much different (*M*_n_ = 60,000), but the molecular weight distribution was broad (2.1). **NBE-EN** was also polymerized by the catalytic systems **1** and **1**/**PA** in CH_2_Cl_2_ (Table 1, Entries 25 and 26). The reactions turned quantitative within minutes, and the polymers obtained were of high molecular weight (almost 10-times higher, compared to the above-mentioned catalytic systems) and a narrow molecular weight distribution. In the absence of solvent, the reaction was also quantitative (Entry 27), but a much longer reaction time was required. The molecular weight of **PNBE-EN** formed also increased greatly, approaching 1,000,000. In this case, the molecular weight distribution was very broad, but the error was large, as the molecular weight was very high, outside the calibration curve regime. NMR spectroscopy was very useful and informative with respect to the nature of the polymer formed. The absence of peaks at 32–15 ppm in the ^13^C-NMR spectrum (Supplementary Material Figure S6b) confirmed that no polymer was formed via the ionic polymerization route (Supplementary Material Scheme S2). The presence of peaks at 5.06–5.50 ppm in the ^1^H-NMR spectrum (Supplementary Material Figure S6a) revealed that the C–C double bond of the norbornene moiety was activated, while the other double bond was inert.

Norbornene derivatives bearing silyl groups have been used for the synthesis of polymeric membranes and can be polymerized via the metathesis or via the insertion polymerization mechanism [30]. Both alkoxy-silyl-norbornenes that we studied (**NBE-SiM** and **NBE-SiE**) were activated by **1** in CH_2_Cl_2_ and provided ROMP polymers (as evidenced by the ^1^H-NMR spectrum; Supplementary Material Figure S7) in moderate yields (Entries 27 and 28). The addition of **PA** (Entry 29) improved both the rate and the yield of the reaction. Interestingly, in THF, the reaction proceeded via the insertion polymerization pathway (as shown by the ^1^H-NMR spectrum; Supplementary Material Figure S8), but provided very small amounts (6%) of polymer. The molecular weights of the polymers obtained could not be measured, because of the negative refraction index of the solution. Similar reactivity (to **1** or **1**/**PA** in CH_2_Cl_2_) has been observed with both heterogeneous and homogeneous Re-, Ru- and W-based catalytic systems [31].

The calculation of the *cis* content of the soluble substituted polynorbornenes was not possible, because of the broad peaks of the olefinic protons due to overlapping signals of *endo*- and *exo*-isomers and the presence of HH, HT and TT units [26,32,33], as well as the complexity of the ^13^C-NMR spectra.

Finally, the polymerization of **DCPD** was studied under various reaction conditions, as this is a reaction of both academic and industrial interest. **DCPD** is an inexpensive and readily available monomer and provides industrial polymers (**PDCPD**) of high mechanical strength [11,34]. The reaction of **1** with **DCPD** in CH_2_Cl_2_ and toluene, as well as in the absence of solvent provided low yields of insoluble **PDCPD** (Table 1, Entries 33–35). Reaction in CH_2_Cl_2_ proceeded in a similar fashion at several **1**/**DCPD** molar ratios, ranging from 1/300–1/1000; in toluene ratios higher than 1/500, it provided traces of polymer, while in the bulk ratios up to 1/500, it gave optimum yields. The addition of **PA** turned the reaction quantitative in CH_2_Cl_2_ and toluene, yielding insoluble polymers (Entries 36 and 37). In the bulk, traces of insoluble **PDCPD** were obtained (Entry 38). **DCPD** is known to provide insoluble polymers, because of extensive crosslinking due to secondary metathesis or radical reactions on the double bond of the cyclopentene ring [35]. Thermogravimetric analysis of **PDCPD** samples obtained by the aforementioned systems showed a single decomposition peak at 470 °C (Supplementary Material Figure S9), which confirms the high degree of crosslinking [34].

### 2.3. Cationic Polymerization of **CPD**

Cyclopentadiene (**CPD**) is an inexpensive cyclic diene, which is usually polymerized via cationic polymerization. Its polymers are characterized by low Tg’s, although they feature rigid cyclic repeat units with one double bond in the main chain. Polymerization of **CPD** by **1** was studied in CH_2_Cl_2_, THF and toluene, as well as without solvent (Table 1, Entries 39–42). The reaction proceeded with low or very low yield in the last three cases and provided low molecular weight polymers. In CH_2_Cl_2_, the yield was high, and the molecular weight of **PCPD** formed had increased. The molar ratio of **CPD**/**1** was equal to 250/1. At higher ratios, the reactions were slower and provided only oligomers. The polymer obtained in all cases came from cationic processes. That was evidenced by the analysis of ^1^H- and ^13^C-NMR data (Supplementary Material Figure S10) [36]. Both 1,2- and 1,4-isomers were formed (Supplementary Material Figure S2), with the 1,4-isomer prevailing (~60%) in all cases. The addition of **ΡΑ** (molar ratios of **ΡΑ**/**1** ranged from 2/1–30/1) did not affect the nature or the stereoselectivity of the reaction (Entries 43 and 44) and provided oligomeric or low molecular weight **PCPD**. That behavior was rather expected, as the nature of the polymerization is not metathetic. **1** is not the first bimetallic compound that has been utilized for the polymerization of **CPD**. Multiply-bonded homogeneous or silica-immobilized dimolybdenum ({Mo_2_}^4+^; δ^2^π^4^δ^2^) *co*mpounds, *i.e*., [Mo_2_(μ-O_2_CMe)_2_(NCMe)_6_](BF_4_)_2_ and [Mo_2_(NCMe)_8_](BF_4_)_4_ [36], have been also studied and exhibited similar reactivity with **1**, under analogous reaction conditions, although the molecular characteristics of the polymers obtained were not reported.

### 2.4. Mechanistic Considerations

The mechanistic study of non-well-defined catalytic systems has always been an intriguing problem, as isolation and characterization of the active intermediates (metallocyclobutanes or metallocarbenes) is in many cases not possible. The small initiation efficiencies of most catalytic systems and/or the high sensitivity of the active species are the most frequent limitations. However, several studies of Mo- or W-based catalytic systems have been published, based mostly on NMR spectroscopic data [14,16,37,38,39,40,41,42,43,44].

**Figure 1:**
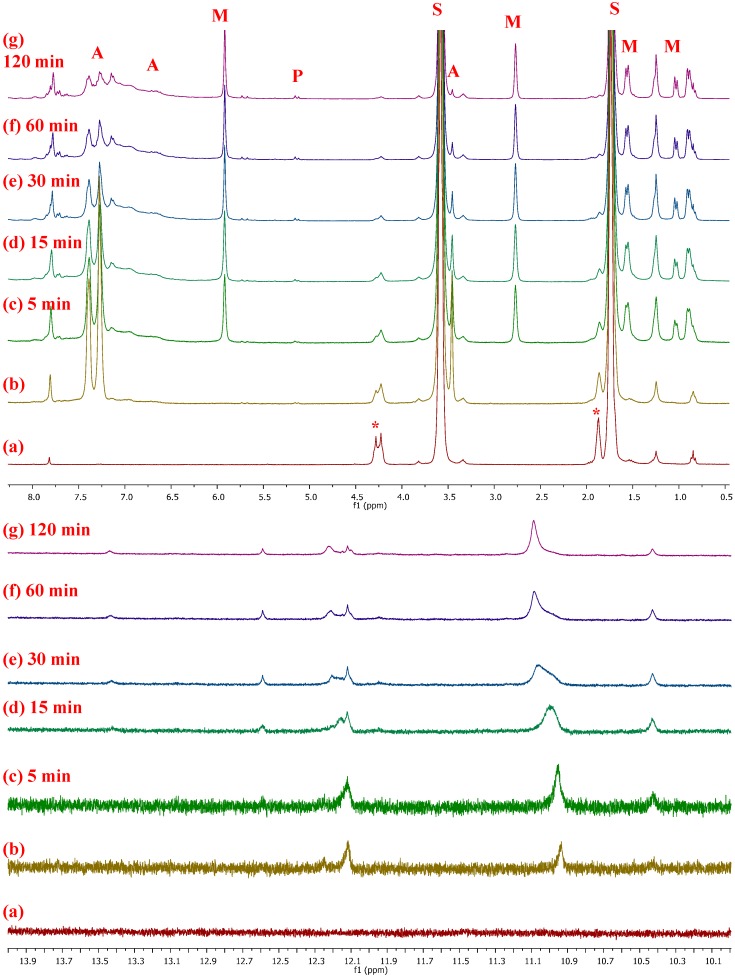
^1^H-NMR spectra (top: region 8.5–0.5 ppm; bottom: region 14.0–10.0 ppm) of: **1** (**a**), from the reaction of **1** (8.0 mg, 0.008 mmol) with **PA** (5 μL, 4.6 mg, 0.05 mmol) (**b**) and from the reaction of **1**/**PA** with **NBE** (5 mg, 0.05 mmol) in *d*^8^-THF at various time intervals, as indicated (**c**–**g**). Signals denoted by “*” are due to coordinated THF of **1**, “S” to residual solvent, “M” to **NBE**, “P” to **PNBE** and “A” to **PA** and/or **PPA**.

In our case, the first stages of **NBE** polymerization by **1**/**PA** were studied by ^1^H-NMR in *d*^8^-THF (Figure 1). The low rate of the reaction in THF allowed for better monitoring of the reaction course. The reaction was carried out at room temperature and with molar ratio of **1**/**PA**/**NBE** equal to 1/6/6. Upon addition of **PA** to a solution of **1** in *d*^8^-THF, a number of peaks appeared in the W-carbene region (Figure 1; bottom), with those at 10.93 and 12.12 ppm predominating. The same peaks were observed in our previous study on the polymerization of **PA** by **1** [16], in which the presence of at least two active alkylidene propagating centers was documented. In the present study, those peaks increased over time, even after the addition of **NBE**. However, after the initiation of **NBE** polymerization, the intensity of the aforementioned peaks decreased, and new peaks emerged, at 11.09 and 12.16 ppm, which represented the active propagating alkylidene species, in agreement with our previous studies on the polymerization of **NBE** by **1** [14]. Quenching the reaction mixture with benzaldehyde (**1**/**NBE**/PhCHO: 1/6/12, 10 μL) caused the disappearance of the high-field peaks. The formation of several alkylidenes and the complex multistage nature of the polymerization reactions induced by **1** render assignment of the peaks mentioned above very difficult; however, more detailed studies as well as theoretical calculations are in progress.

## 3. Experimental Section

### 3.1. General

Starting materials were purchased from Sigma-Aldrich (Sigma-Aldrich, St. Louis, MO, USA) and are of the highest available purities. Complex Na[W_2_(µ-Cl)_3_Cl_4_(THF)_2_]·(THF)_3_ (**1**) [15] was prepared according to literature procedures. **NBE** was dissolved in the solvent used for the reaction, was dried by stirring with CaH_2_ under argon and was distilled under vacuum prior to use. **NBD** was passed through an Al_2_O_3_ column. **PA** and **DCPD** were dried by stirring with CaH_2_ under argon, distilled under vacuum and stored in the dark under argon. **CPD** was obtained by distillation of **DCPD** (retro Diels-Alder reaction) immediately before use. All monomers were checked for purity by GC-MS and ^1^H-NMR spectroscopy. THF and Et_2_O were distilled over Na/Ph_2_CO, toluene and hexanes over Na, CH_2_Cl_2_ over CaH_2_ and methanol over sodium methoxide. Benzaldehyde was purified by distillation under reduced pressure. All solvents were distilled in an inert atmosphere and were degassed by three freeze-pump-thaw cycles, with the exception of methanol, which was degassed by bubbling nitrogen or argon for 0.5 h. All operations were performed under a pure dinitrogen or argon atmosphere, using Schlenk techniques on an inert gas/vacuum manifold or in a dry box (O_2_, H_2_O < 1 ppm).

NMR spectra were recorded on a Varian Unity Plus 300 spectrometer (Varian, Palo Alto, CA, USA). In all cases, chemical shifts are reported in ppm relative to the deuterated solvent resonances. Size exclusion chromatography (SEC) experiments were carried out with a modular instrument consisting of a Waters Model 600 pump (Waters, Milford, MA, USA), a Waters Model U6K sample injector, a Waters Model 410 differential refractometer and a set of 4 μ-Styragel columns with a continuous porosity range of 10^6^–10^3^ Å. The columns were housed in an oven thermostated at 40 °C. THF was the carrier solvent at a flow rate of 1 mL/min. The instrument was calibrated with PS standards covering the molecular weight range of 4000–900,000. The thermal stability of the polymers was studied by thermogravimetric analysis (TGA), employing a Q50 TGA model from TA instruments. Samples were placed in platinum crucibles. An empty platinum crucible was used as a reference. Samples were heated from ambient temperatures to 600 °C in a 60-mL/min flow of N_2_ at a heating rate of 10 °C/min.

### 3.2. Catalytic Reactions

A typical procedure is described as follows. **PA** (e.g., 9.2 mg, 0.09 mmol) was added to a solution of **1** (**1**: 9.0 mg, 0.009 mmol) in a solvent (2.0 mL), followed by the substrate (e.g., **NBE**, 423 mg, 4.5 mmol). When no **PA** was used, the substrate was added to the solution of **1**. The mixture was allowed to react at room temperature for a given time (Table 1), after which it was concentrated to half volume and treated with an excess of methanol to have the polymeric products precipitated. The resulting solids were filtered and washed repeatedly with methanol. They were redissolved in THF, and the above procedure was repeated at least three times. The products were dried *in vacuo*.

### 3.3. Catalytic Reactions in NMR Tubes

Complex **1** was dissolved in *d*^8^-THF (8.0 mg, 0.008 mmol), and the green solution was transferred in an NMR tube. The appropriate amounts of **PA** (5 μL, 4.6 mg, 0.05 mmol) and **NBE** (5 mg, 0.05 mmol) were added using a microliter syringe.

### 3.4. Polymer Microstructure

The stereochemistry of the polymers obtained in this study was determined by ^1^H- and ^13^C-NMR [1,18,20,26,32,36].


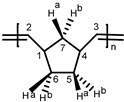
**PNBE.**
^1^H-NMR (CDCl_3_, 300 MHz): 5.25 (s, 2H, H^2,3^
*t*), 5.10 (s, 2H, H^2,3^
*c*), 2.73 (br, s, 2H, H^1,4^
*c*), 2.37 (br, s, 2H, H^1,4^
*t*), 1.95–1.60 (br, m, 3H, H^5a,6a,7a^), 1.50–1.20 (br, m, 2H, H^5b,6b^), 1.20–0.85 ppm (br, m, 1H, H^7b^); ^13^C-NMR (CDCl_3_, 75.4 MHz): 133.99 (s, C^2,3^
*ccc*), 133.21 (m, C^2,3^
*ctt/ttt/ctc*), 133.08 (s, C^2,3^
*ttc*), 43.67 (s, C^1,4^
*tc*), 43.44 (s, C^1,4^
*tt*), 42.88 (s, C^7^
*cc*), 42.25 (s, C^7^
*ct/tc*), 41.52 (s, C^7^
*tt*), 38.88 (s, C^1,4^
*cc*), 38.67 (s, C^1,4^
*ct*), 33.39 (s, C^5,6^
*cc*), 33.19 (s, C^5,6^
*ct*), 32.61 (s, C^5,6^
*tc*), 32.43 ppm (s, C^5,6^
*tt*).
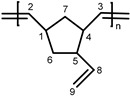
**PVNBE.**
^1^H-NMR (CDCl_3_, 300 MHz): 5.76 (br, 1H, H^8^), 5.30 (br, 2H, H^2,3^), 4.89–4.96 (br, 2H, H^9^), 2.19–3.10 (br, 3H, H^1,4,5^), 1.10–2.10 ppm (br, m, 2H, H^6,7^); ^13^C-NMR (CDCl_3_, 75.4 MHz): 141.7, 140.6 (s, C^8^), 135.6–130.3 (m, C^2,3^), 113.6, 113.1 (s, C^9^), 50.1, 47.9 (s, C^5^),45.6, 37.5 (s, C^1^), 45.6, 41.3 (s, C^4^), 42.8, 41.3, 39.5 (s, C^7^), 41.3 ppm (s, C^6^).
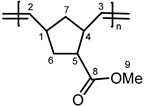
**PNBE-COOME.**
^1^H-NMR (CDCl_3_, 300 MHz): 5.26–5.37 (br, 2H, H^2,3^), 2.76–3.10 (br, s, 1H, H^4^), 2.26 (br, s, 1H, H^5^), 1.80–2.10 (br, m, 3H, H^9^), 1.10–1.60 ppm (br, m, 3H, H^1,6,7^); ^13^C-NMR (CDCl_3_, 75.4 MHz): 170.7 (s, C^8^), 140.9–127.4 (m, C^2,3^), 51.1 (s, C^9^), 48.6–34.8 ppm (s, C^1,4,5,6,7^).
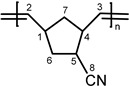
**PNBE-CN.**
^1^H-NMR (CDCl_3_, 300 MHz): 5.15–5.75 (2H, H^2,3^), 3.52 (1H, H^4^), 2.80–3.35 (1H, H^1^), 2.00–2.68 (br 1H, H^5^), 1.40–1.95 (br, 2H, H^6^), 1.10–1.40 and 2.50–2.56 ppm (2H, H^7^); ^13^C-NMR (CDCl_3_, 75.4 MHz): 125.0–140.0 (C^2,3^), 120.0–125.0 (C^8^), 32.5–48.5 ppm (C^1,4,5,6,7^).
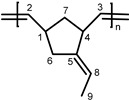
**PNBE-EN.**
^1^H-NMR (CDCl_3_, 300 MHz): 5.06–5.50 (br, m, 3H, H^2,3,8^), 2.86 (br, s, 2H, H^1,4^
*c*), 2.50 (br, s, 2H, H^1,4^
*t*), 1.80–2.20 (br, m, 2H, H^6^), 1.57 (s, 3H, H^9^), 1.10–1.30 ppm (br, s, 2H, H^7^); ^13^C-NMR (CDCl_3_, 75.4 MHz): 146.0 (C^5^
*t*), 145.6 (C^5^
*c*), 134.6 (C^2^
*t*, *TH*), 134.5 (C^2^
*c*, *TH*), 134.0 (C^2^
*c*, *TT*), 133.7 (C^2^
*t*, *TT*), 132.9 (C^3^
*c*,*t*, *HH*), 132.4 (C^3^
*c*, *HT*), 132.0 (C^3^
*t*, *HT*), 117.0 (C^8^
*t*), 116.2 (C^8^
*c*), 48.9 (C^4^
*t*), 44.2 (C^4^
*c*), 42.9–42.7 (C^7^), 41.8 (C^6^), 38.1–36.4 (C^1^), 14.9–14.0 ppm (C^9^).
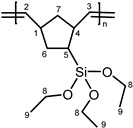
**PNBE-SiE (ROMP).**
^1^H-NMR (CDCl_3_, 300 MHz): 5.10–5.80 (br, m, 2H, H^2,3^), 3.85 (br, s, 6H, H^8^), 1.40–2.20 (br, m, 7H, H^1,4,5,6,7^), 1.22 ppm (br, s, 9H, H^9^); ^13^C-NMR (CDCl_3_, 75.4 MHz): 130.0–140.0 (C^2,3^), 58.4 (C^8^), 40.0–50.0 (C^1,4,6,7^), 26.0–27.0 (C^5^), 18.2 ppm (C^9^).
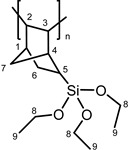
**PNBE-SiE (cationic).**
^1^H-NMR (CDCl_3_, 300 MHz): 3.60–3.90 (br, m, 6H, H^8^), 1,30–2,00 (br, 9H, H^1,2,3,4,5,6,7^), 1.10–1.30 ppm (m, 9H, H^9^); ^13^C-NMR (CDCl_3_, 75.4 MHz): 58.8 (C^8^), 18.8 ppm (C^9^).
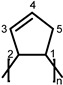

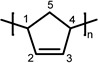
**PCPD 1,2.**
^1^H-NMR (CDCl_3_, 300 MHz): 5.50–5.75 (Η^3,4^), 2.62 (Η^5^), 2.42 ppm (Η^1,2^); ^13^C-NMR (CDCl_3_, 75.4 MHz): 133.04 (C^3^), 130.03 (C^4^), 55.09 (C^2^), 44.70 (C^1^), 36.40 ppm (C^5^).
**PCPD 1,4.**
^1^H-NMR (CDCl_3_, 300 MHz): 5.50–5.75 (H^2,3^), 2.02 (H^1,4^), 1.62 ppm (H^5^); ^13^C-NMR (CDCl_3_, 75.4 MHz): 134.17 (C^2,3^), 50.53 (C^1,4^), 32.03 ppm (C^5^).

## 4. Conclusions

From this work the following conclusions can be drawn:

(a) The **1**/**PA** catalytic system catalyzes the ROMP of norbornene (**NBE**) and a number of substituted norbornenes efficiently, providing high molecular weight polymers in high yields and high stereoselectivity (86% *cis* for **PNBE**). Monomers bearing strongly-coordinating pendant groups (–COOH, –OH, –CN) were either not activated by **1**/**PA** (the first two) or provided polymers in low yields (case of –CN), while those bearing weaker ones (–COOMe, –CH=CH_2_, =CHCH_3_) showed high reactivity. It should be noted that less strained double bonds remained unaffected (–CH=CH_2_, =CHCH_3_). Norbornadiene (**NBD**) and dicyclopentadiene (**DCPD**) were also activated quickly and quantitatively, giving insoluble, highly crosslinked polymers.
(b) **1** activated cyclopentadiene (**CPD**), but in a different fashion, providing oligomers or low molecular weight polymers, not via metathesis, but via the cationic polymerization pathway.
(c) Compared to **1**, the catalytic system **1**/**PA** was in general more active towards the ROMP of all monomers studied, in all solvents, as well as in bulk. The molecular weights of the polymers obtained were higher (with very few exceptions), while the molecular weight distributions were either retained or improved. The *cis* specificity of **PNBE** was the same (86% *cis*) with either system.
(d) *In situ* monitoring of the reaction (**1**/**PA**/**NBE**) by ^1^H-NMR spectroscopy revealed the formation of active alkylidenes of the propagating chains, in agreement with our previous studies, but a detailed mechanistic study of the catalytic system is underway.

## Supplementary Materials

Supplementary materials can be accessed at: http://www.mdpi.com/1420-3049/20/12/19810/s1.
