# Supplementary material for: Exploring the Reactivity of Na[W2(μ-Cl)3Cl4(THF)2]∙(THF)3 towards the Polymerization of Selected Cycloolefins
Source: Molecules. 2015 Dec 8;20(12):21896–908. doi: 10.3390/molecules201219810 (PMC6332418; doi:10.3390/molecules201219810)
Supplement: Supplementary file 1 [file molecules-20-19810-s001.pdf]

Supporting Information: Exploring the Reactivity of $Na[W_2(\mu\text{-}Cl)_3Cl_4(THF)_2]\cdot(THF)_3$ towards the Polymerization of Selected Cycloolefins

Nikolaos Saragas, Georgios Floros, Grigorios Raptopoulos, Marinos Pitsikalis, Patrina Paraskevopoulou and Konstantinos Mertis

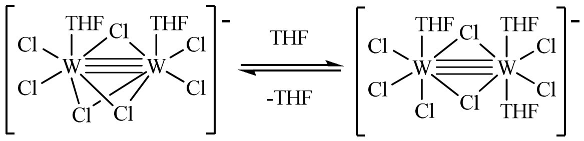

1 **1'**

Scheme S1. Schematic representation of complex **1** and the equilibrium between **1** and **1′**.

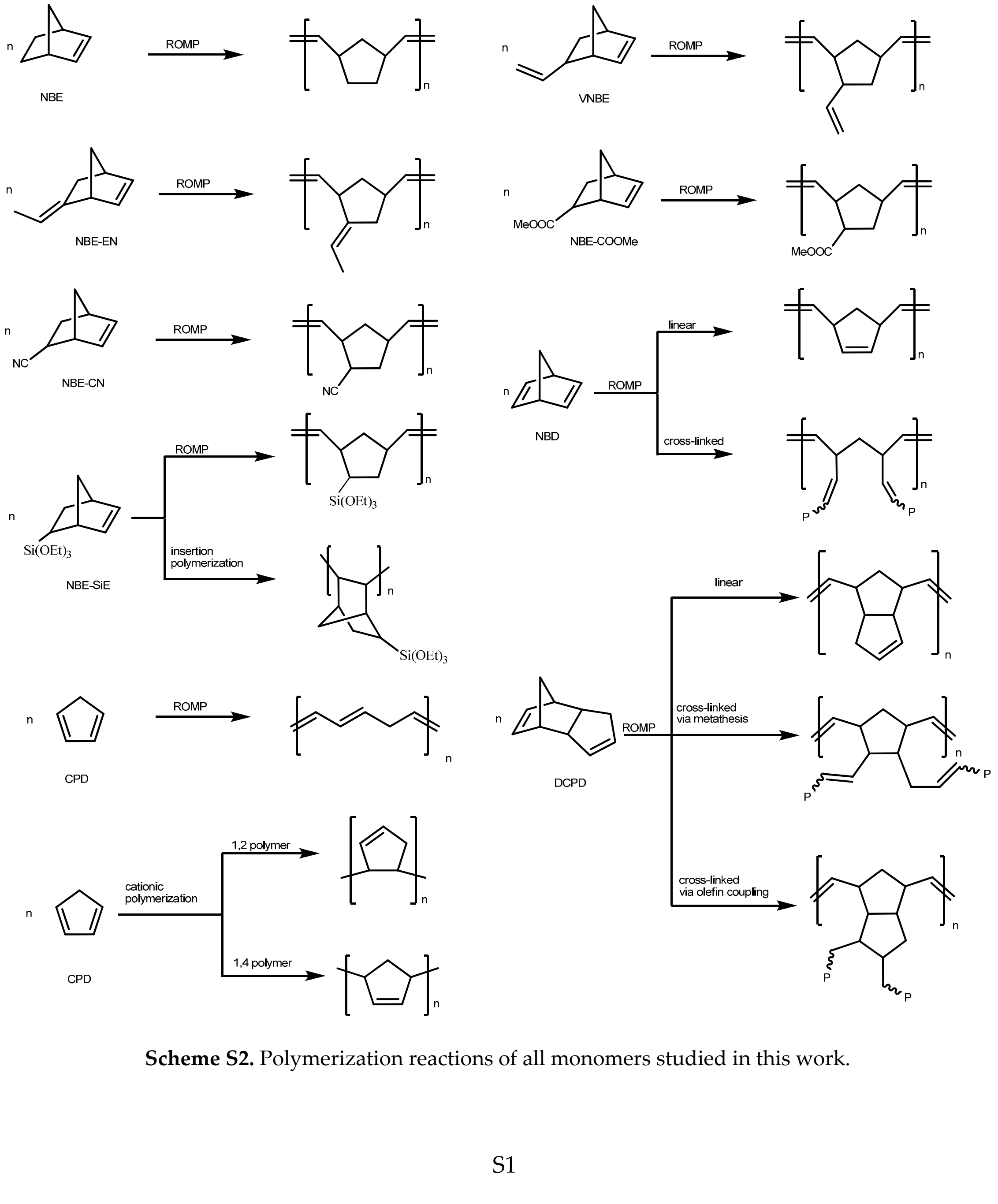

Scheme S2. Polymerization reactions of all monomers studied in this work.

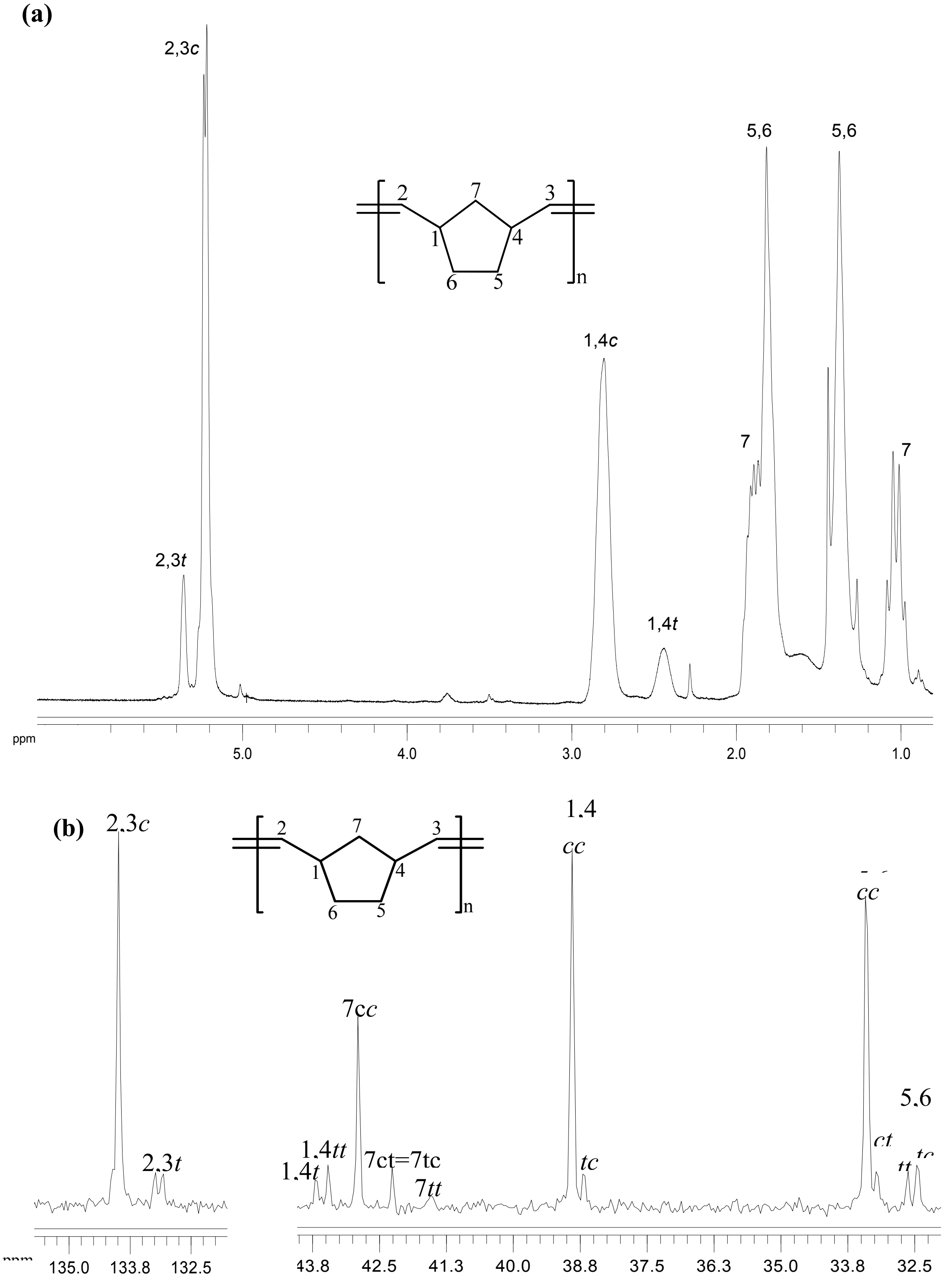

Figure S1. (**a**) ^{1}H- and (**b**) ^{13}C{^{1}H}-NMR spectra ($CDCl_3$) of **PNBE** obtained from the reaction of **1/PA/NBE** in CH_2Cl_2.

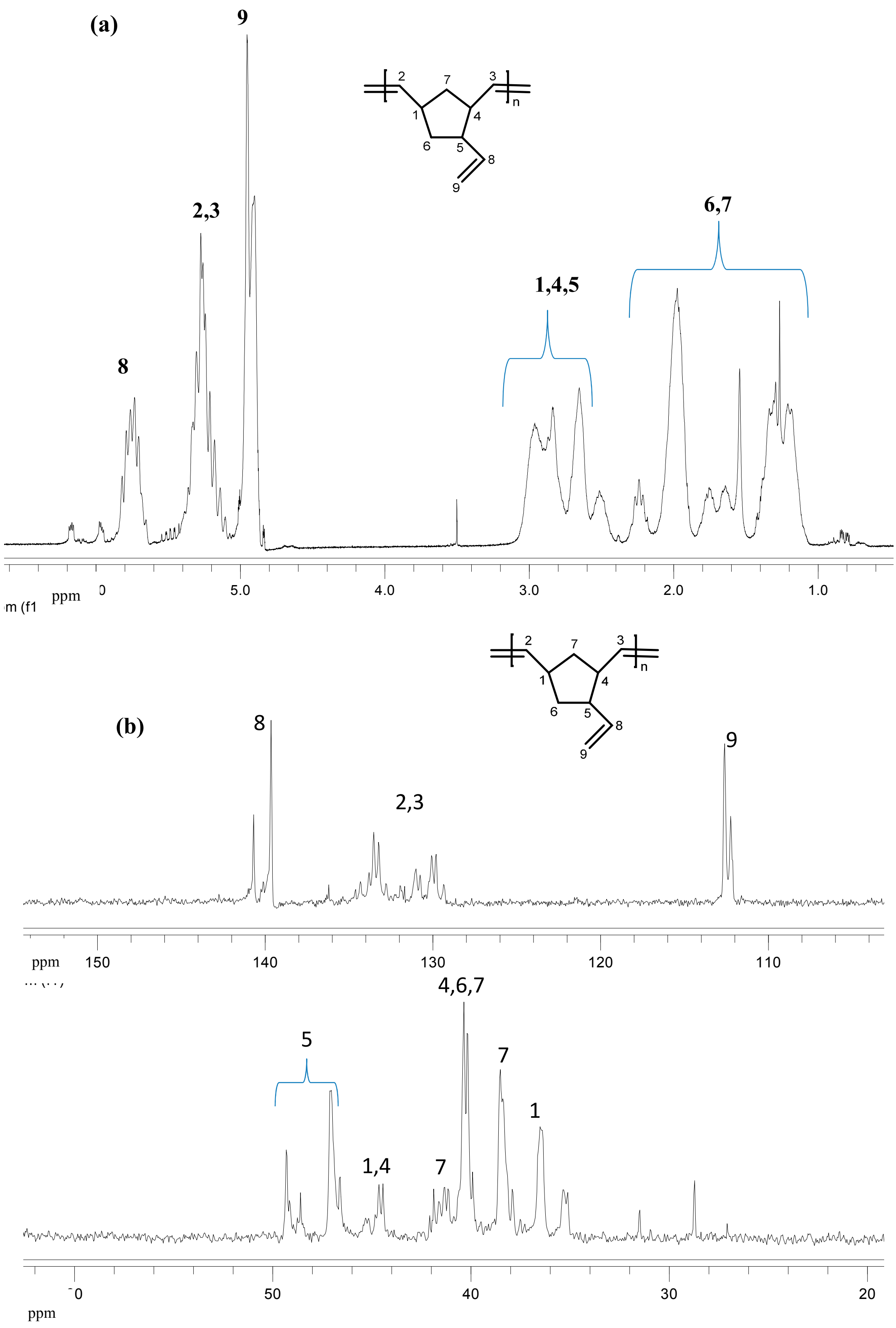

Figure S2. (**a**) ^{1}H- and (**b**) $^{13}C\{^{1}H\}$-NMR spectra ($CDCl_3$) of **PVNBE** obtained from the reaction of **1/PA/VNBE** in CH_2Cl_2.

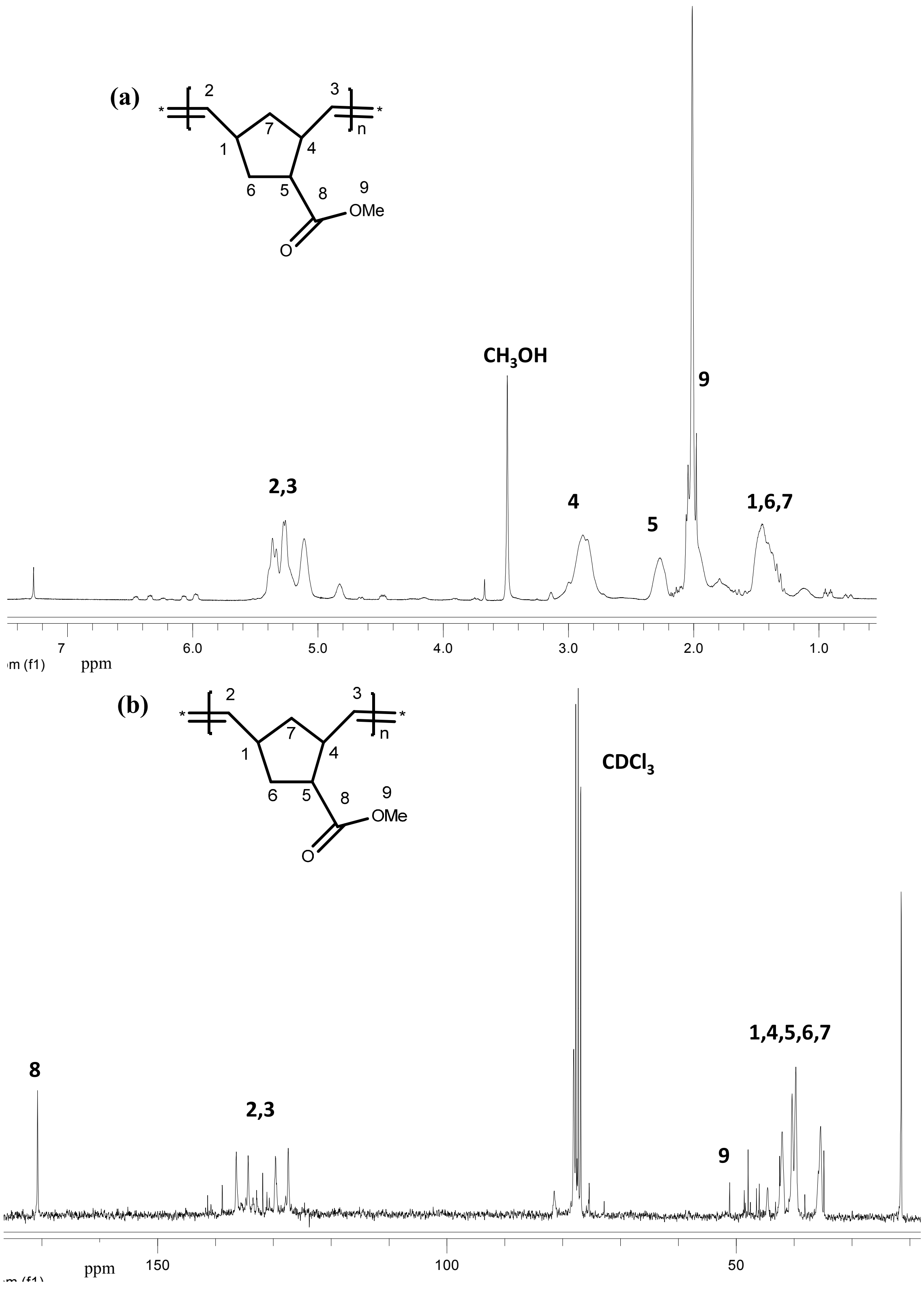

Figure S3. (**a**) ^{1}H- and (**b**) $^{13}C\{^{1}H\}$-NMR spectra ($CDCl_3$) of **PNBE-COOMe** obtained from the reaction of **1/PA/NBE-COOMe** in CH_2Cl_2.

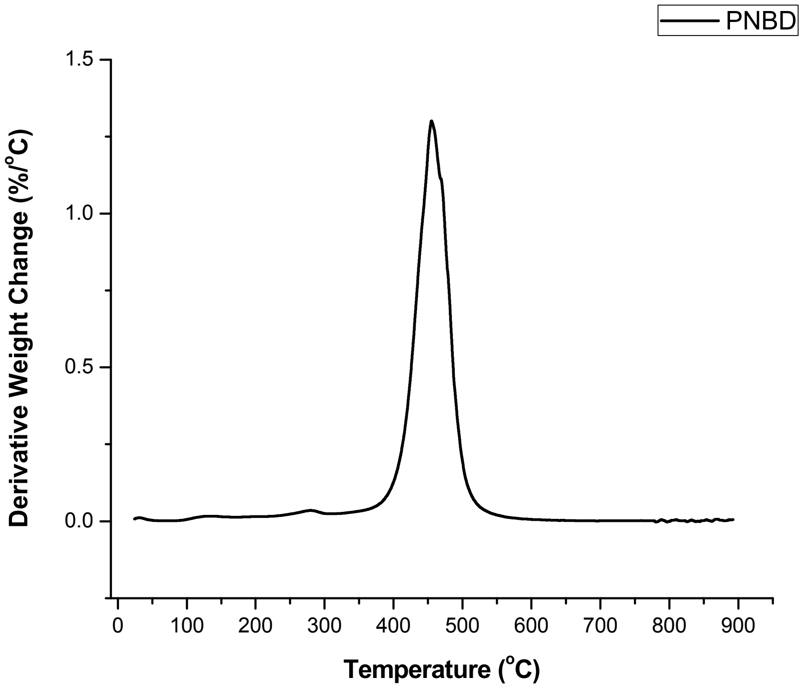

Figure S4. Derivative weight change with temperature of **PNBD** obtained from the reaction of **1/PA/NBD** in THF.

(a)

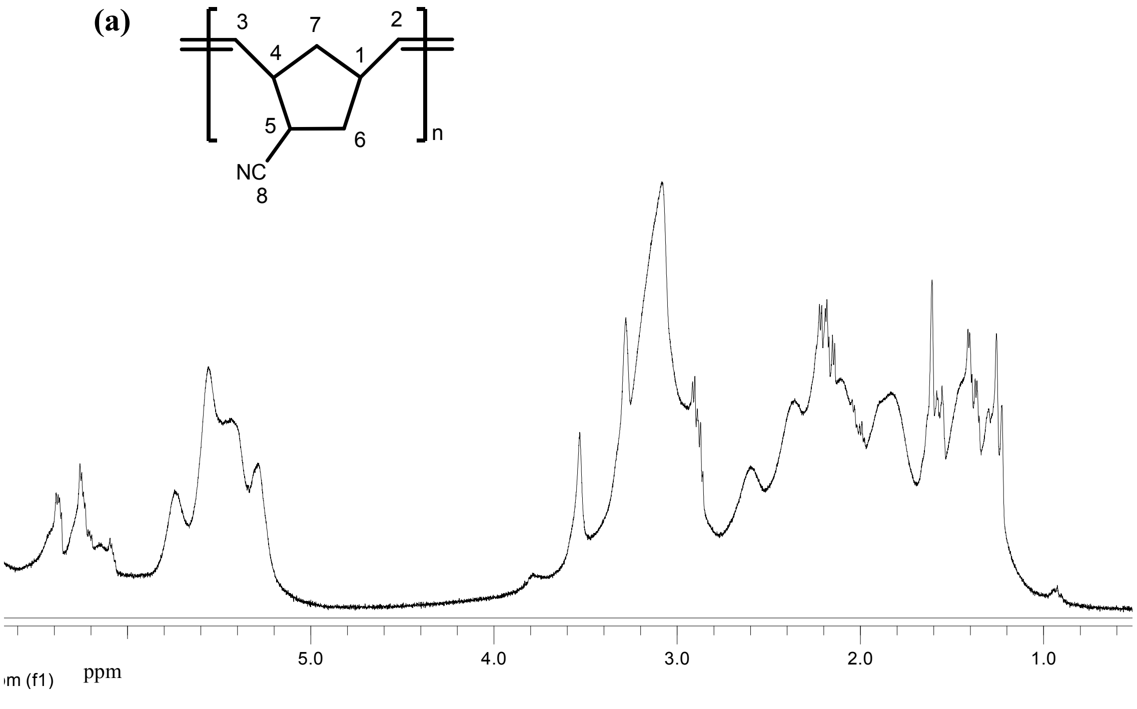

Figure S5. *Cont.*

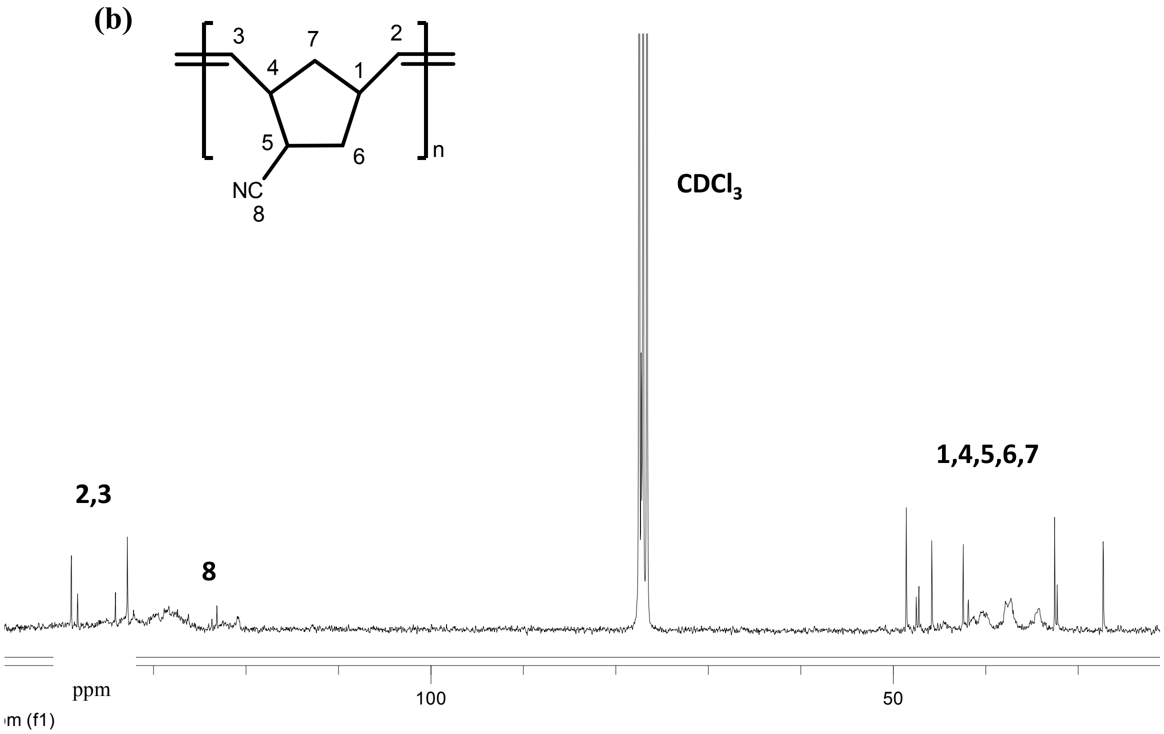

Figure S5. (**a**) ^{1}H- and (**b**) ^{13}C{^{1}H}-NMR spectra ($CDCl_3$) of **PNBE-CN** obtained from the reaction of **1/PA/NBE-CN** in CH_2Cl_2.

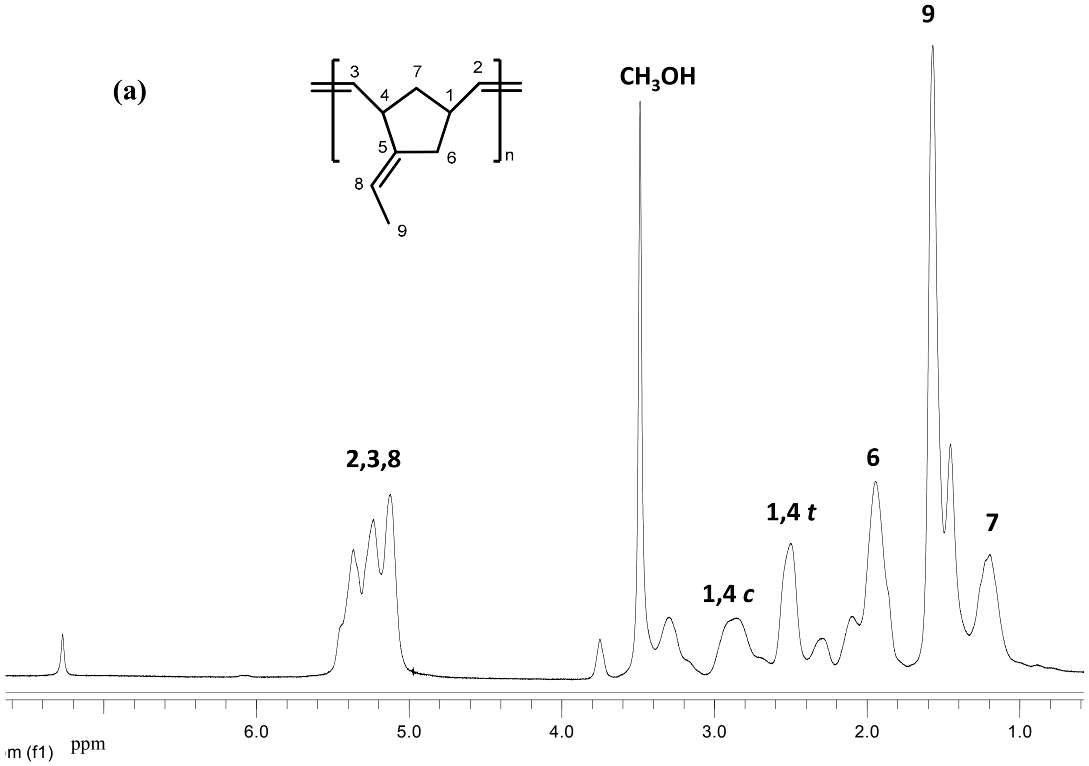

Figure S6. *Cont.*

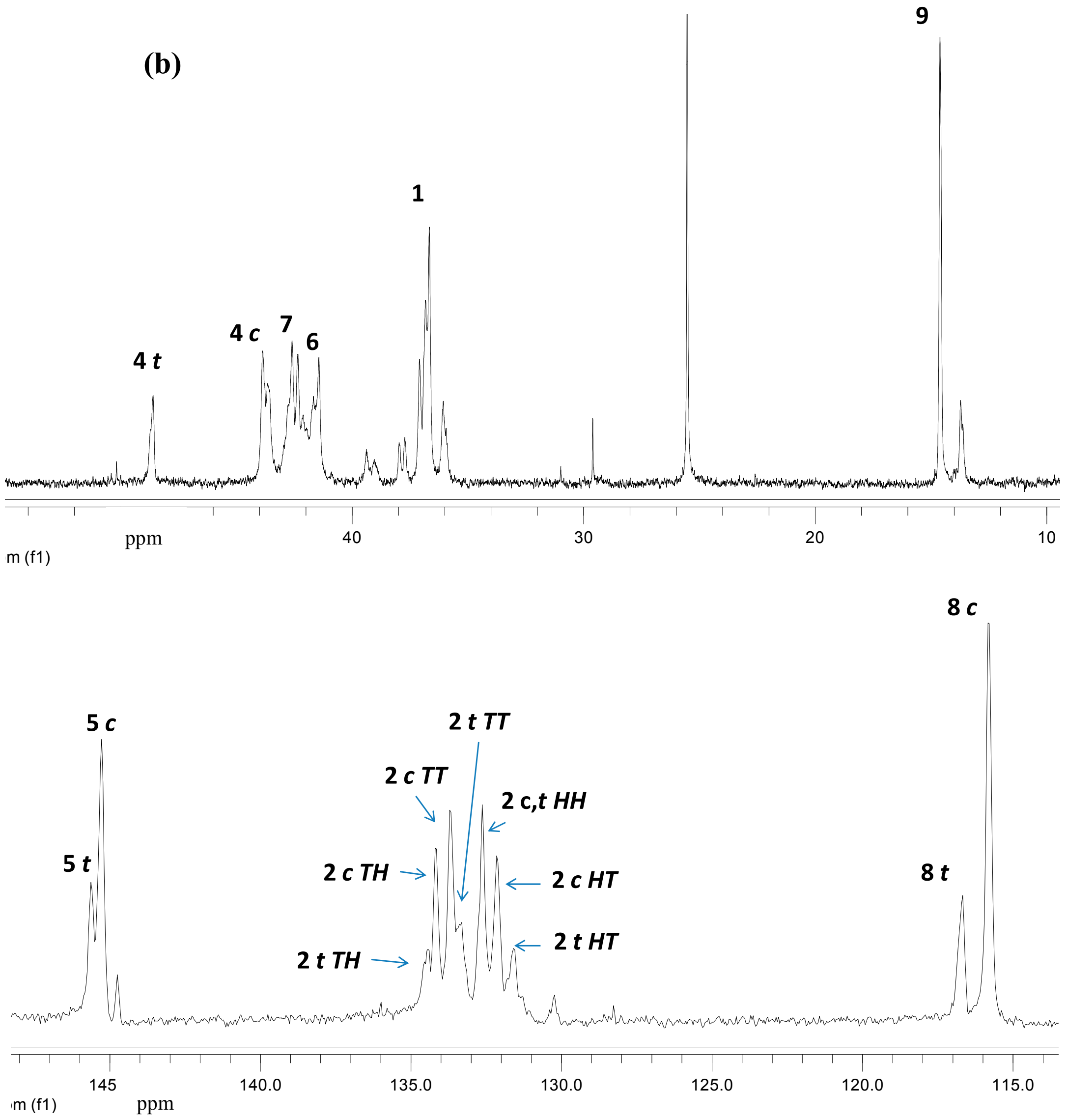

Figure S6. (**a**) ^{1}H- and (**b**) $^{13}C\{^{1}H\}$-NMR spectra ($CDCl_3$) of **PNBE-EN** obtained from the reaction of **1/PA/NBE-EN** in CH_2Cl_2.

(a)

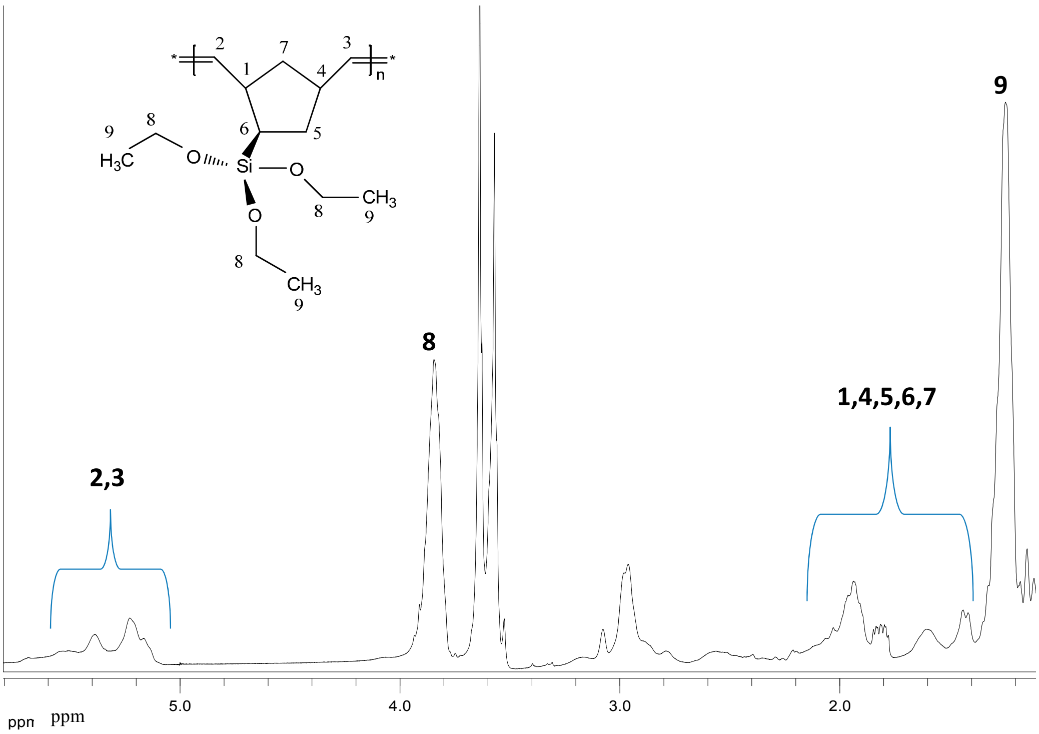

(b)

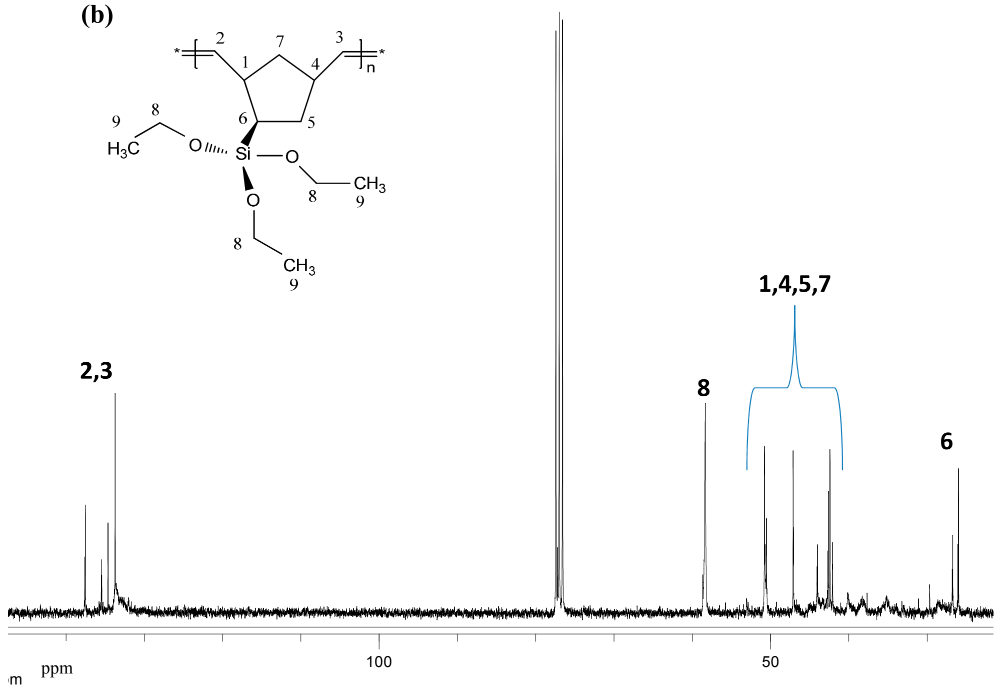

Figure S7. (**a**) ^{1}H- and (**b**) $^{13}C\{^{1}H\}$-NMR spectra ($CDCl_3$) of **PNBE-SiE** obtained from the reaction of **1/NBE-SiE** in CH_2Cl_2.

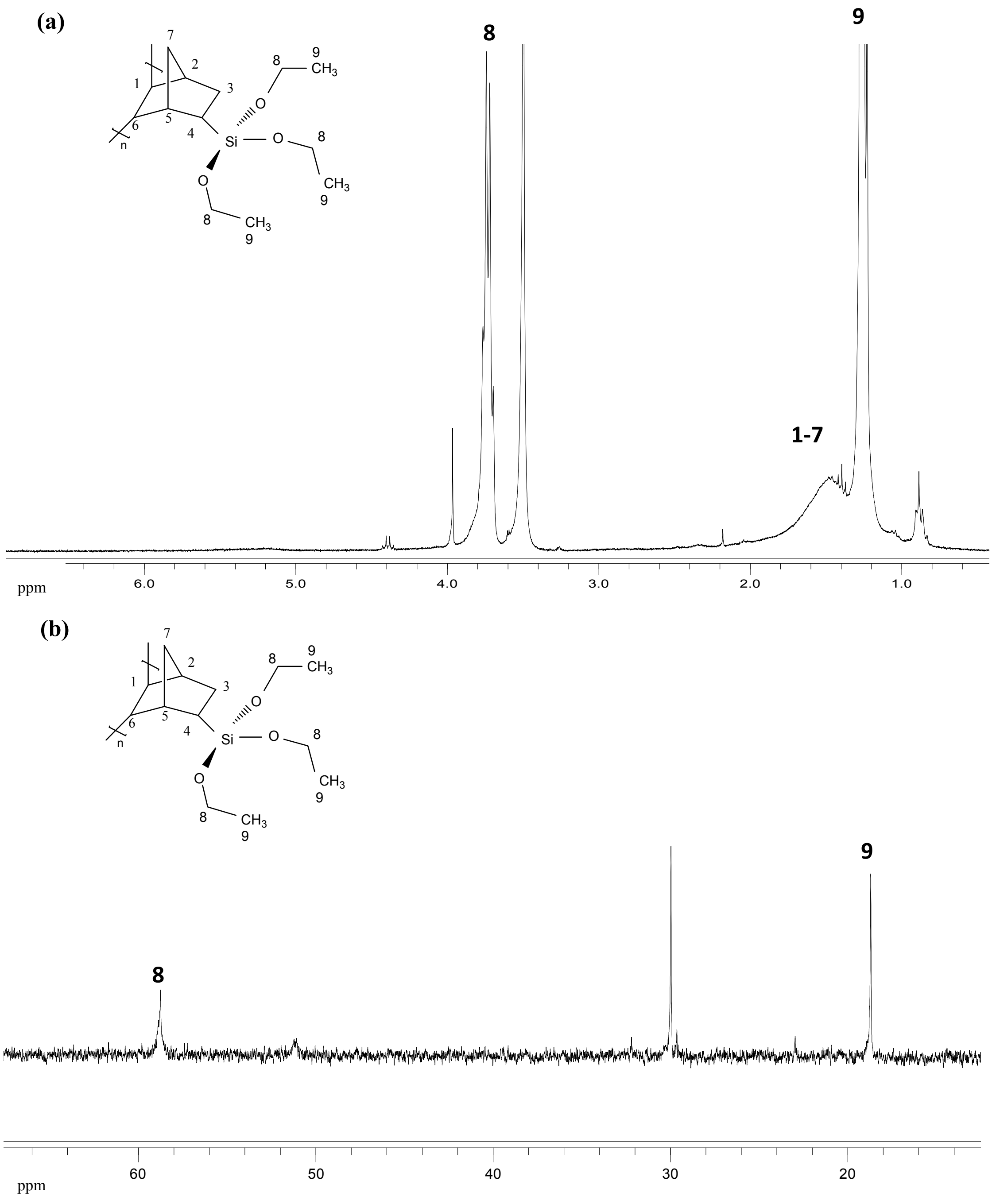

Figure S8. (**a**) ^{1}H- and (**b**) ^{13}C{^{1}H}-NMR spectra ($CDCl_3$) of **PNBE-SiE** obtained from the reaction of **1** and **NBE-SiE** in THF.

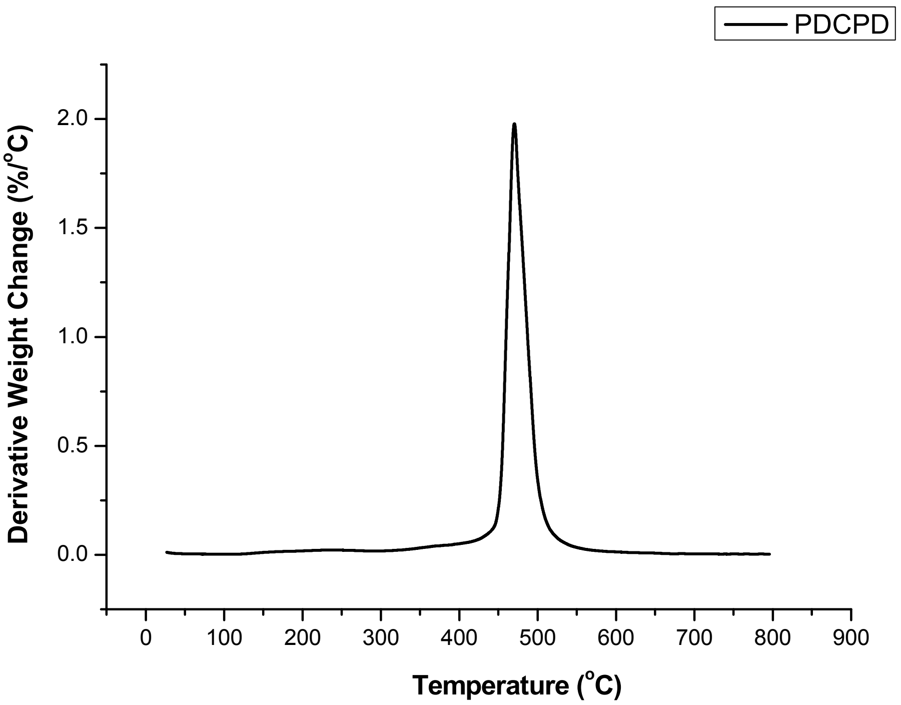

Figure S9. Derivative weight change with temperature of insoluble **PDCPD** obtained from the reaction of **1/PA/DCPD** in CH_2Cl_2.

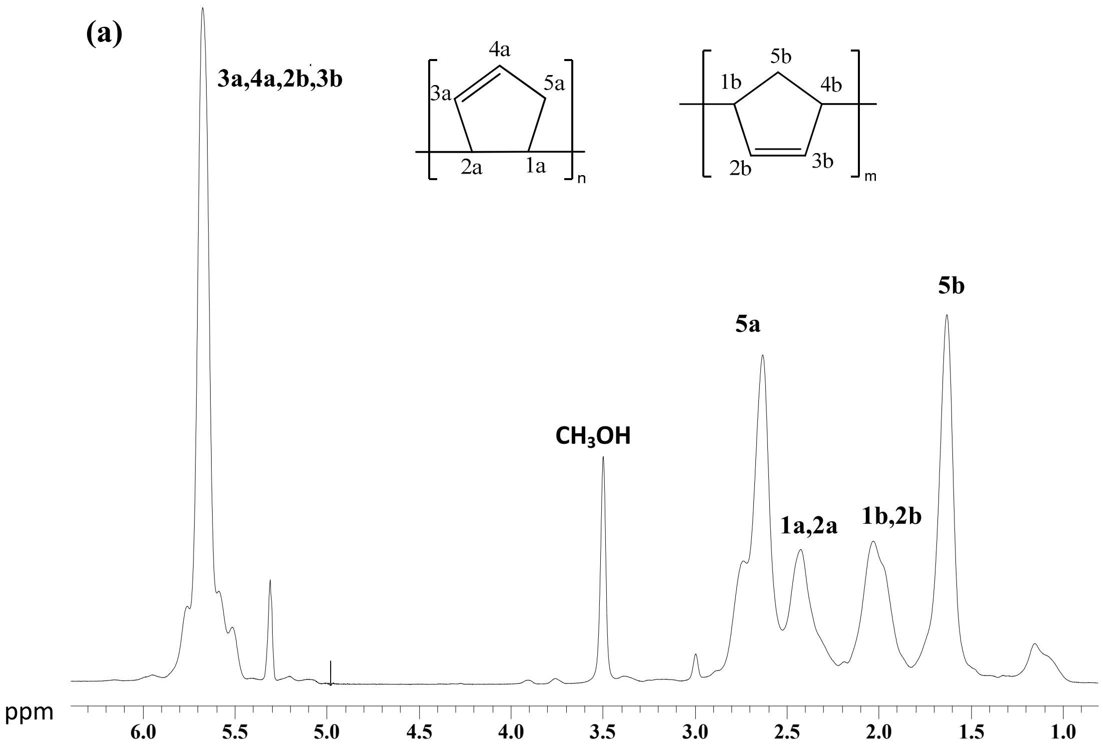

Figure S10. *Cont.*

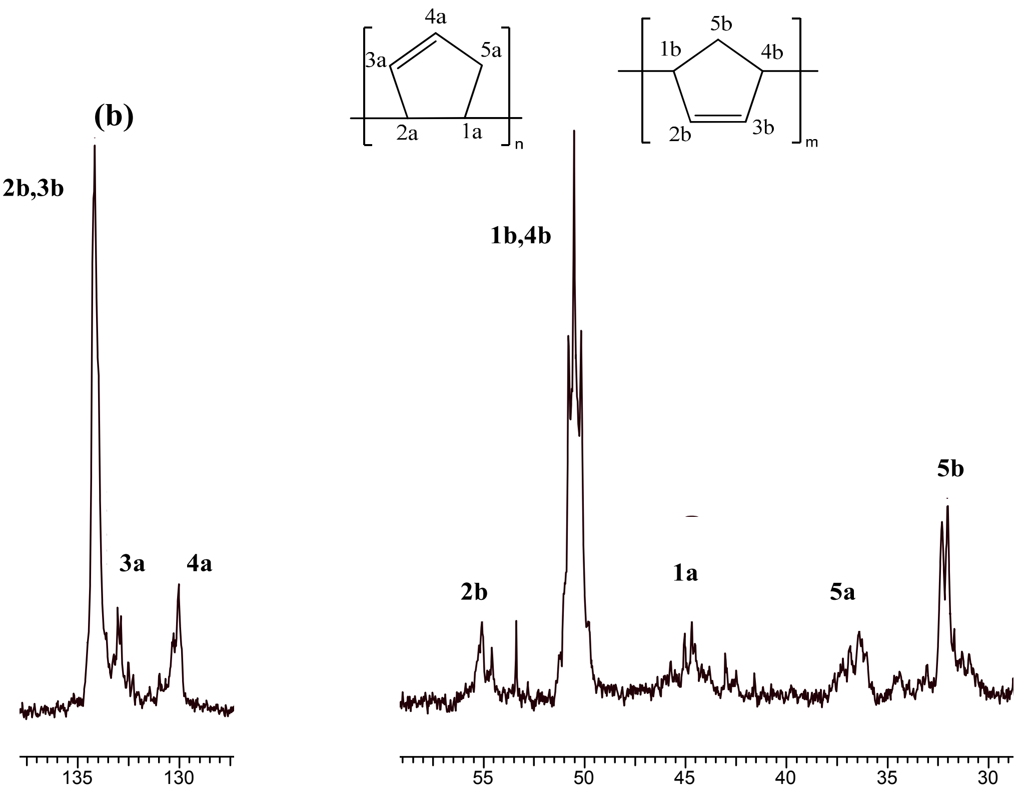

Figure S10. (**a**) ^{1}H- and (**b**) $^{13}C\{^1H\}$-NMR spectra ($CDCl_3$) of **PCPD** obtained from the reaction of **1/CPD** in CH_2Cl_2.
